# Metastatic Squamous Cell Carcinoma in a Northern Brown Bandicoot (*Isoodon macrourus*)

**DOI:** 10.3390/vetsci4010010

**Published:** 2017-02-14

**Authors:** Amanda P. Beck, Amy L. Shima, Mark D. Bennett, Linda K. Johnson

**Affiliations:** 1 College of Public Health, Medical and Veterinary Sciences, James Cook University, Townsville 4811, Queensland, Australia; amanda.beck@einstein.yu.edu (A.P.B.); dvm.shima@gmail.com (A.L.S.); linda.k.johnson@ucdenver.edu (L.K.J.); 2 School of Veterinary and Life Sciences, Murdoch University, South Street Campus, Murdoch 6150, Western Australia, Australia

**Keywords:** neoplasia, northern brown bandicoot, *Isoodon macrourus*, squamous cell carcinoma, immunohistochemistry, epithelial to mesenchymal transition

## Abstract

Aside from a handful of notable exceptions, neoplasia is not reported as a major cause of mortality in wild animal populations and often goes undetected. For northern brown bandicoots specifically, there are few reported tumors in the literature and on file in the Australian Registry of Wildlife Health. This report describes a case of squamous cell carcinoma in a northern brown bandicoot (*Isoodon macrourus*), with metastases to the draining lymph nodes and lung. This neoplasm consisted predominantly of well-differentiated squamous cells and multifocal keratin pearls, with areas possibly consistent with epithelial to mesenchymal transition, as identified by positive immunohistochemical staining by both pancytokeratin (AE1/AE3) and vimentin. Additional investigations were negative for bandicoot papillomatosis carcinomatosis viruses.

## 1. Introduction

Neoplasia is typically not reported as a major cause of mortality in wild animal populations and often goes undetected due to the difficulties associated with evaluating health and disease status in wildlife [1,2,3]. Notable exceptions to this rule of thumb include the Devil Facial Tumour Disease of Tasmanian Devils (*Sarcophilus harrisii*), sea turtle fibropapillomatosis, and genital carcinoma of California Sea Lions (*Zalophus californianus*) [3]. Reported cases of neoplasia in peramelids in the literature and on file in the Australian Registry of Wildlife Health include a variety of mesenchymal and epithelial tumors [4,5]. However, perhaps the most well-studied neoplasms of bandicoot species are those of the cutaneous and mucocutaneous papillomatosis and carcinomatosis syndrome, which is associated with the infection of the western barred bandicoot (*Perameles bougainville*) by bandicoot papillomatosis carcinomatosis virus type 1 (BPCV1), and a similar syndrome in southern brown bandicoots (*Isoodon obesulus*) associated with the closely related BPCV2 [3,6,7,8,9,10,11].

The northern brown bandicoot (*Isoodon macrourus*) is considered to be one of Australia’s most common bandicoots [12]. Reported tumors in *I. macrourus* include lymphosarcoma, mammary adenocarcinoma, and metastatic pulmonary adenocarcinoma foci [4]. This report describes a case of metastatic squamous cell carcinoma (SCC) in *I. macrourus*, which, to the authors’ knowledge, is the first report of this tumor type in this species.

## 2. Materials and Methods

A complete post-mortem examination was conducted, and grossly visible lesions were identified, described, and photographed as necessary. Tissues were fixed in 10% buffered formalin, processed routinely for 4 µm sectioning, and then stained with hematoxylin and eosin (H&E). Immunohistochemistry (IHC) of the neoplasm was performed using anti-pancytokeratin (clone AE1/AE3, 1:75, Dako Australia) and anti-vimentin (clone V9, 1:400, Dako Australia) antibodies. Staining was performed on sections of tissue from the same paraffin block, and processing, antigen retrieval, endogenous peroxidase quenching, primary and secondary antibody incubation times, counterstaining, and controls were performed as previously described [13]. To probe for evidence of papillomavirus and BPCV infection within the neoplasm, IHC and in situ hybridization (ISH) were performed as previously described [7,9]. Positive control tissue for ISH came from BPCV2-positive formalin-fixed paraffin-embedded *I. obesulus* skin.

## 3. Results

### 3.1. Animal Details

A 1.4 kg adult female *I. macrourus* was submitted to the pathology service at the James Cook University School of Veterinary and Biomedical Sciences. The animal had been accidentally live-trapped in Tarzali, northern Queensland (17°25’31”S; 145°36’13”E) and was subsequently presented to a wildlife veterinarian. On physical examination, the animal was subdued and emaciated and there was a large, firm, lobulated mass on the right hindlimb. Based on the clinical presentation, the decision was made to humanely euthanize the animal with intravenous pentobarbitone sodium (Lethabarb®, Virbac, Australia).

### 3.2. Post-Mortem Examination

**Figure 1:**
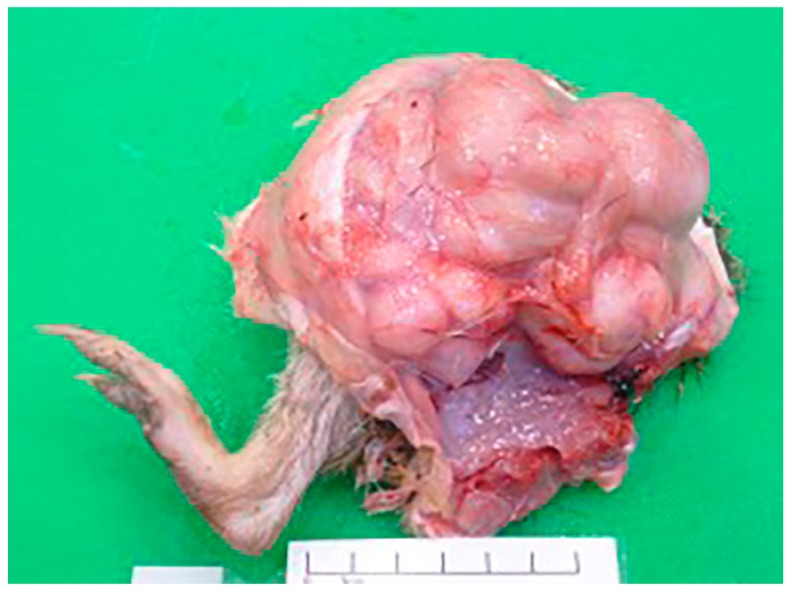
A smooth, pale tan, multilobulated mass circumferentially surrounds the right femoral-tibial joint of an adult female *I. macrourus*.

At post-mortem examination, the *I. macrourus* was thin, with prominent vertebral bodies and minimal subcutaneous adipose tissue. A 7 cm × 8 cm × 8 cm firm, pale tan, smooth, multilobulated mass, weighing 415 g, circumferentially encircled the femoral-tibial joint of the right hindlimb (Figure 1). The mass contained pockets of blood-stained turbid fluid admixed with areas of fibrosis and necrosis. Multifocally, there was superficial lateral ulceration of the mass. The right inguinal lymph node was diffusely and mildly enlarged. Multiple irregular, slightly raised, beige masses (0.5 to 1 cm in diameter) elevated the visceral pleura of the right and left lung lobes.

### 3.3. Histopathology

**Figure 2:**
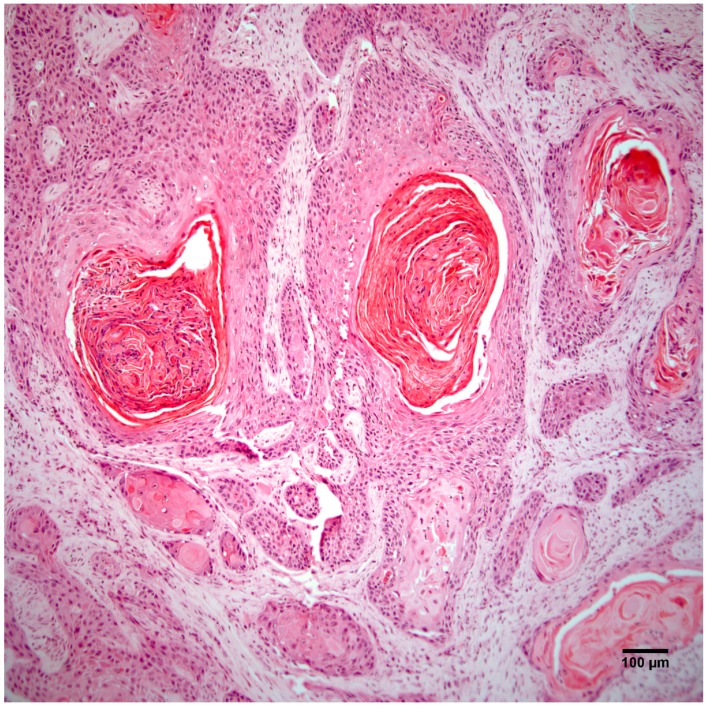
The right hindlimb mass is an unencapsulated dermal and subcutaneous neoplasm composed of nests, streams, and sheets of neoplastic cells that multifocally surround central accumulations of keratin. Hematoxylin and eosin staining. Scale bar 100 µm.

**Figure 3:**
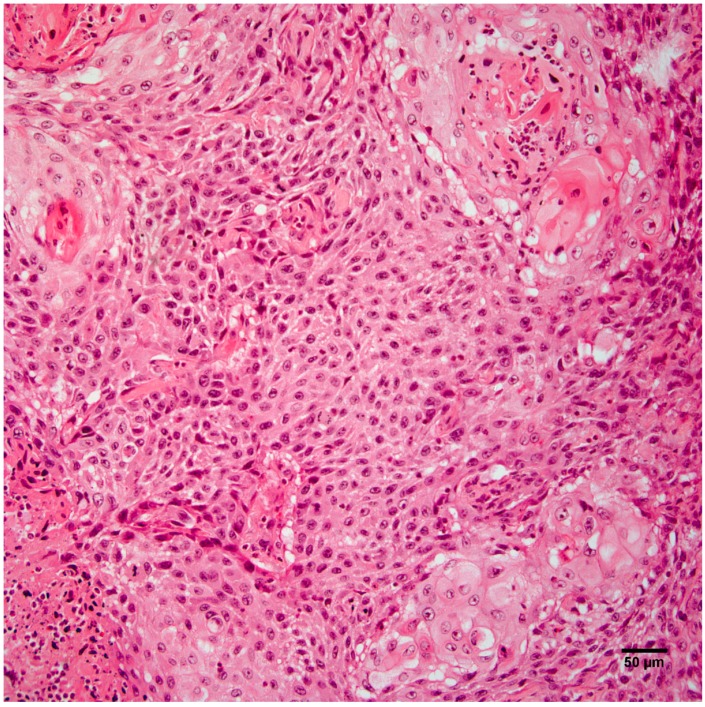
Neoplastic cells from the right hindlimb mass are polygonal to fusiform and occasionally display intercellular bridging. Hematoxylin and eosin staining. Scale bar 50 µm.

Histopathology of the leg mass revealed an unencapsulated dermal mass extending widely within the subcutis and composed of nests, streams, and sheets of neoplastic cells interspersed between collagen bundles. Multifocally, neoplastic cells surrounded centralized accumulations of eosinophilic lamellated material (keratin) (Figure 2). Neoplastic cells were polygonal to fusiform with a moderate amount of eosinophilic cytoplasm and occasionally displayed intercellular bridging (Figure 3). There was moderate anisocytosis and anisokaryosis and mitoses (0–2 per high power field) were sometimes bizarre. Within the mass were frequent areas of necrosis and foci of basophilic angular refractile material (mineralization). Examination of the grossly identified lung nodules revealed sheets, streams, and multifocal whorls of the above described neoplastic cells. The normal architecture of inguinal and peri-renal lymph nodes was effaced and replaced by sheets of neoplastic cells and keratin pearls similar to those in the dermal and pulmonary masses. Histologic features of the leg mass, as well as those in the lung and lymph nodes, were consistent with squamous cell carcinoma (SCC).

### 3.4. Immunohistochemistry and In Situ Hybridization

**Figure 4:**
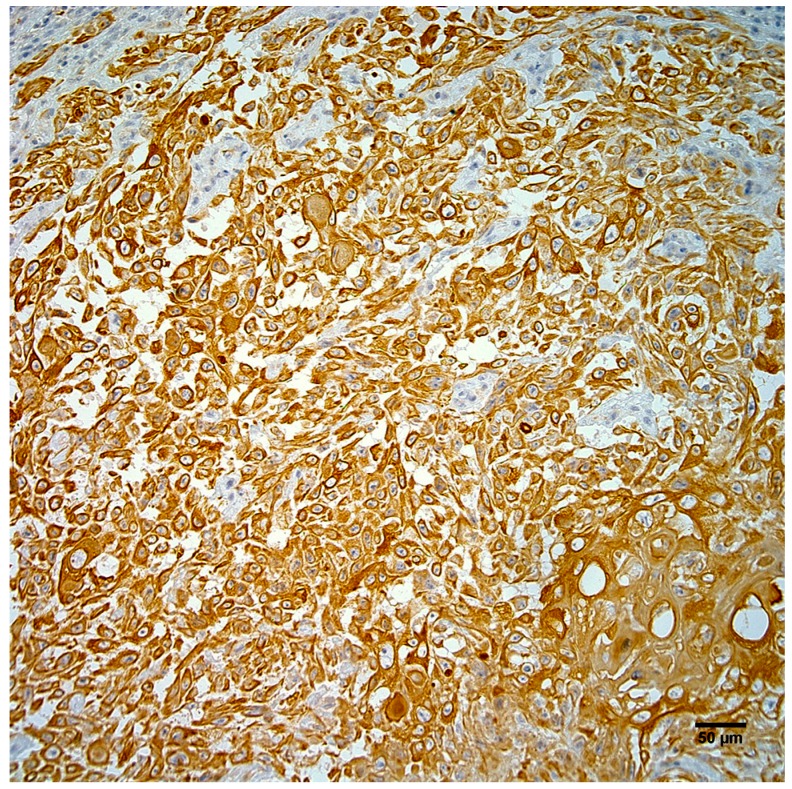
Neoplastic cells from the right hindlimb mass exhibit strong positive cytoplasmic immunostaining for pancytokeratin. Hematoxylin counterstain. Scale bar 50 µm.

**Figure 5:**
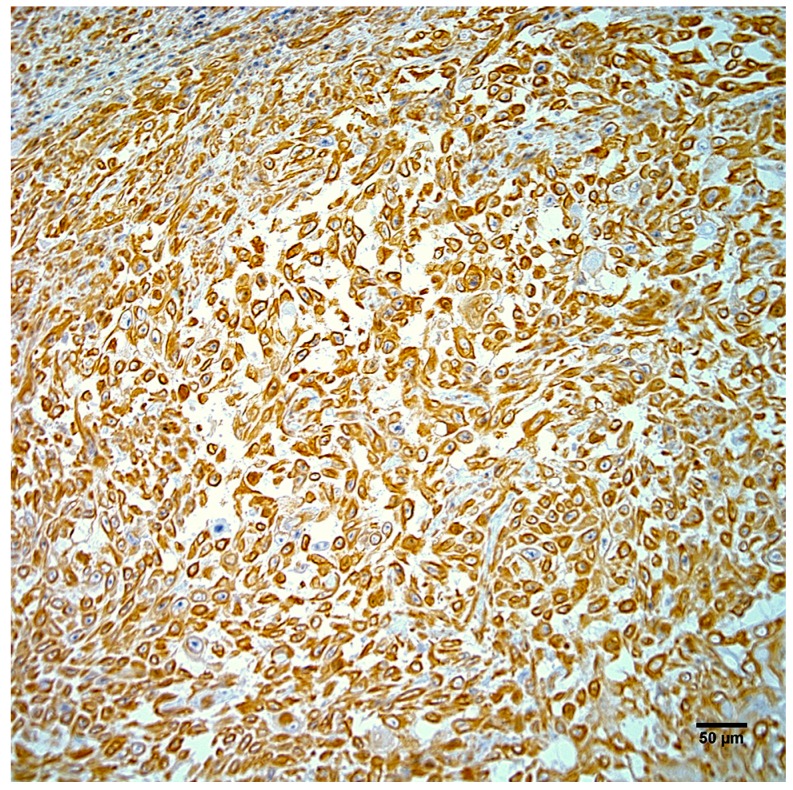
Neoplastic cells from the right hindlimb mass exhibit strong positive cytoplasmic immunostaining for vimentin. Hematoxylin counterstain. Scale bar 50 µm.

Neoplastic cells exhibited strong positive cytoplasmic staining for intermediate filaments of pancytokeratin (Figure 4) and vimentin (Figure 5). Immunohistochemistry did not demonstrate papillomavirus or BPCV L1 protein in the test sections and ISH also failed to demonstrate any BPCV2-like DNA in the neoplasm.

## 4. Discussion

This report describes a case of metastatic SCC in *I. macrourus*. Previously, there have been only sporadic reports of neoplasia in *I. macrourus*, and to the authors’ knowledge, this is the first report of this tumor type in this species. In general, neoplastic lesions are infrequently reported in bandicoots, with the exception of those linked to BPCVs 1 and 2. Infection with these viruses is associated with a progressively debilitating papillomatosis and carcinomatosis syndrome in western barred and southern brown bandicoots, respectively [3,6,7,8,9,10,11]. Though it is possible a similar condition exists in *I. macrourus*, there was no histologic or molecular evidence of viral involvement in the case of SCC presented here. However, previous experience with BPCV1 in *P. bougainville* showed that the most histologically malignant lesions were the least likely to have demonstrable ISH positivity [6,11]. This trend was also evident in skin swab PCR evidence that showed a reduction in the percentage of positive results in more dysplastic lesions [10]. Therefore, despite the failure to detect BPCV-like DNA or L1 protein, there still exists the possibility that a virus may have initiated the squamous cell neoplastic process in this case. Ideally, samples from similar lesions in future cases will be collected and fixed in both glutaraldehyde and formalin, with additional tissue retained fresh or frozen for appropriate molecular tests.

An interesting feature of the neoplasm in this report was the presence of areas of the tumor that stained positively for both pancytokeratin and vimentin intermediate filaments, which is potentially consistent with the phenomenon of epithelial to mesenchymal transition (EMT). EMT is a process that converts epithelial cells into mesenchymal cells [14,15,16]. Although it is considered physiological during implantation, embryogenesis, and wound repair, it can play a pathological role in epithelial cancer progression and metastasis [14,15,16].

EMT is largely controlled at the transcriptional and translational level in response to various extracellular signals in the local microenvironment [14,15,17]. These signals include hypoxia, inflammation, and soluble growth factors including transforming growth factor beta, fibroblast growth factor, epidermal growth factor, hepatocyte growth factor/scatter factor, insulin-like growth factor, and platelet-derived growth factor [14,15,16,17]. EMT cells lose their epithelial phenotypic characteristics (polygonal shape, apical-basal polarity, expression of typical epithelial cell markers) and become spindle shaped, expressing vimentin, smooth muscle actin, desmin, fibroblast-specific protein 1, fibronectin, and/or stromelysin-3, as is typical of mesenchymal cells [14,15,16,17,18].

A possible alternate classification for the neoplastic lesions described in this case is biphasic synovial sarcoma with scattered foci of squamous differentiation, however we consider this differential diagnosis to be unlikely. Synovial sarcomas are malignant mesenchymal tumors with variable epithelial differentiation that despite their name, do not to arise from synovium [19]. Typically, the epithelial component of biphasic synovial sarcomas is glandular; only approximately 1% of these tumors in humans exhibits squamous metaplasia with keratinization [19].

## 5. Conclusions

In conclusion, this case report describes a SCC in a wild *I. macrourus* that exhibited draining lymph node and pulmonary metastasis as well as evidence to suggest epithelial to mesenchymal transition. Though this tumor type has not previously been reported in this species, it should be considered as a differential diagnosis for wild or captive *I. macrourus* presenting with dermal masses and/or disseminated disease. Efforts to link the tumor to BPCV2, a virus from *I. obesulus* associated with papillomas and metastatic SCC, failed in this case. However, future investigations of similar presenting skin lesions could undertake a more complete array of molecular techniques on unfixed tissue samples to test for BPCV-like viruses.
